# Long-Term Cognitive and Behavioral Outcomes following Resolution of Sleep Disordered Breathing in Preschool Children

**DOI:** 10.1371/journal.pone.0139142

**Published:** 2015-09-29

**Authors:** Sarah N. Biggs, Lisa M. Walter, Angela R. Jackman, Lauren C. Nisbet, Aidan J. Weichard, Samantha L. Hollis, Margot J. Davey, Vicki Anderson, Gillian M. Nixon, Rosemary S. C. Horne

**Affiliations:** 1 The Ritchie Centre, Hudson Institute of Medical Research, Melbourne, Australia; 2 Department of Paediatrics, Monash University, Melbourne, Australia; 3 Melbourne Children’s Sleep Centre, Monash Children’s, Monash Medical Centre, Melbourne, Australia; 4 Melbourne School of Psychological Sciences, The University of Melbourne, Melbourne, Australia; 5 Clinical Sciences Research, Murdoch Children’s Research Institute, Melbourne, Australia; Oasi Institute for Research and Prevention of Mental Retardation, ITALY

## Abstract

This study aimed to determine the long term effects of resolution of SDB in preschool children, either following treatment or spontaneous recovery, on cognition and behavior. Children diagnosed with SDB at 3-5y (N = 35) and non-snoring controls (N = 25), underwent repeat polysomnography (PSG) and cognitive and behavioral assessment 3 years following a baseline study. At follow-up, children with SDB were grouped into Resolved and Unresolved. Resolution was defined as: obstructive apnea hypopnea index (OAHI) ≤1 event/h; no snoring detected on PSG; and no parental report of habitual snoring. 57% (20/35) of children with SDB received treatment, with SDB resolving in 60% (12/20). 43% (15/35) were untreated, of whom 40% (6/15) had spontaneous resolution of SDB. Cognitive reduced between baseline and follow-up, however this was not related to persistent disease, with no difference in cognitive outcomes between Resolved, Unresolved or Control groups. Behavioral functioning remained significantly worse in children originally diagnosed with SDB compared to control children, regardless of resolution. Change in OAHI did not predict cognitive or behavioral outcomes, however a reduction in nocturnal arousals, irrespective of full resolution, was associated with improvement in attention and aggressive behavior. These results suggest that resolution of SDB in preschool children has little effect on cognitive or behavioral outcomes over the long term. The association between sleep fragmentation and behavior appears independent of SDB, however may be moderated by concomitant SDB. This challenges the assumption that treatment of SDB will ameliorate associated cognitive and behavioural deficits and supports the possibility of a SDB phenotype.

## Introduction

Sleep disordered breathing (SDB) affects 12–15% of children [1], with the peak prevalence occurring during the preschool years (ages 3–5 y) [2,3]. SDB ranges in severity from primary snoring (PS), characterized by habitual snoring with no gas exchange abnormalities or increased sleep fragmentation, to obstructive sleep apnea (OSA), characterized by hypoxia, hypercapnia and frequent arousals from sleep [3]. The association between SDB and cognitive and behavioral dysfunction in school-aged children is now well established [4]. Despite its prevalence, less is known about the impact of SDB in younger children. The few studies that have used the gold standard of overnight polysomnography (PSG) to confirm OSA in preschool children, have reported that affected children had significant behavioral deficits [5,6]. The results for cognitive performance are mixed with one study showing no difference in cognitive performance compared to non-snoring children [6] and the other showing deficits in some aspects of cognitive functioning, but not others [7]. The indication that preschool children with SDB present with normal cognitive development yet older children do not leads to a hypothesis that early treatment may ameliorate cognitive deficits developing in later childhood as a result of persistent disease.

SDB in children is predominantly caused by enlarged tonsils and adenoids within a relatively small pharynx, resulting in obstruction of the upper airway during sleep [2,8]. As such, the most common treatment is adenotonsillectomy [9]. The studies assessing the effect of treatment on behavioral functioning have, for the most part, shown a marked improvement in daytime behaviour [10–15]. The effects of treatment on cognitive recovery are however less clear, with some studies showing significant improvements in neurocognition and academic functioning [16,17], but substantially more studies showing little or no change from baseline [13,18–21]. To date, most studies assessing the efficacy of treatment have had follow-up periods of less than one year [5,7,13,16,21–24] or have not compared the results to children with SDB who did not receive treatment [5,7,16,17,21–25]. It is becoming widely accepted that, contrary to early assumptions, PS, the mildest and most common form of SDB, is not benign [26], but carries similar risk for cognitive and behavioral impairment as OSA [27]. Children with PS are often not treated due to lack of sequelae evident on PSG (i.e. hypoxia or elevated sleep fragmentation) and the risks associated with surgery [28], potentially leaving them at risk of continued or even increasing cognitive and behavioral deficit.

To date, there have been limited studies examining the effects of treatment of SDB in preschool children, who are in a critical period for brain development [29,30]. It may be that treating at this earlier age will reduce the risk of future cognitive impairment, either through the resolution of any hypoxia or respiratory related sleep fragmentation, or improvement in behavior, allowing the children to learn more effectively at school. However, previous studies in older children have shown that treatment for SDB is not always effective in resolving sleep and breathing problems [31]. Furthermore, a proportion of those children who are not treated will have spontaneous resolution of SDB [13,18,31]. Thus, the key question is not whether treatment in early childhood improves long-term cognitive and behavioral outcomes in children with SDB, but whether resolution in early childhood, either as a result of treatment or spontaneous recovery, improves long-term outcomes of SDB. Therefore, the aims of this study was to determine: 1. the effectiveness of treatment during the preschool years on the long-term resolution of SDB; and, 2. the effects of resolution, either due to treatment or spontaneous recovery, on cognitive and behavioral outcomes in these children. We hypothesise that 1. a proportion of children with SDB who were treated will have residual disease; and, 2. children with residual disease will experience continued or worsening of cognitive performance and behavioral concerns.

## Methods

Ethical approval for this project was granted by the Monash Health and Monash University Human Research Ethics Committees. Written informed consent was obtained from parents on behalf of the children enrolled in this study after a full explanation of the procedure and the longitudinal nature of the study. Informed assent was obtained verbally from the children and documented on their research record. Written consent was not obtained from the children as they were minors in the legal guardianship of their parents and deemed too young to provide this information. Any child who verbally refused to participate was not enrolled in the study, irrespective of the parent’s written consent. This consent procedure was approved by the above mentioned Human Research Ethics Committees. There was no monetary incentive for participation.

### Subjects

At baseline, 151 children (3–5 y) clinically referred to the Melbourne Children’s Sleep Centre for assessment of SDB and 41 age-matched, non-snoring controls underwent overnight PSG together with neurocognitive and behavioral testing between 2008 and 2011. Control children were recruited from the community via advertisements placed in Monash Medical Centre, Monash University and local newsletters. Children with conditions or taking medications known to affect sleep, breathing, blood pressure, or neurocognitive function were not recruited. Neurocognitive, behavioral, and cardiovascular data from the baseline study have been previously published [6,32–35]. Three years following the baseline study, subjects were invited to return for a follow-up study. Parents of children who did not participate in the follow-up study completed a telephone survey regarding the reasons for non-participation, what treatment, if any, their child had following the baseline study, and current snoring status of their child. The decision to treat was made through consultation between parents and their treating physician as per standard clinical care and was independent of this study.

### Protocol

At the time of both the baseline and follow-up PSG studies, children were otherwise healthy and not undergoing treatment with either nasal steroids or antibiotics. The procedure for both the baseline and follow-up studies were identical. Prior to both PSG studies height and weight were measured and converted to a body mass index (BMI) z-score to adjust for gender and age [36]. Questionnaires relating to demographics, general health, and behavior were completed on the night of the PSG by the parents. Within three weeks of the PSG and prior to commencement of any treatment, participants underwent cognitive assessment in the home, conducted by a trained psychologist who was blinded to the outcomes of the sleep study. The assessments were conducted in a quiet room, separate from the usual activity of the household. One parent was permitted to stay with the child during testing, however they were instructed to remain silent throughout. At this time, parents completed further behavioral, quality of life, and parental stress questionnaires. Parents were also blinded to the results of the follow-up PSG. Results of the quality of life and parental stress questionnaires will not be reported here.

### Polysomnography

Electrophysiological signals were recorded using a commercially available PSG system (E-Series, Compumedics, Melbourne, Australia) and standard pediatric recording techniques.[37] Electrodes for recording electroencephalogram (EEG at baseline: Cz, C4-M1, C3-M2, O2-M1, O1-M2 (≥ 4 y); Cz, C4-M1, C3-M2 (< 4 y); EEG at follow-up: Cz, C4-M1, C3-M2, O2-M1, O1-M2, F4-M1, F3-M2), left and right electrooculogram, submental electromyogram, left and right anterior tibialis muscle electromyogram and electrocardiogram were attached. Thoracic and abdominal breathing movements were detected using respiratory inductance plethysmography (Pro-Tech zRIP™ Effort Sensor, Pro-Tech Services Inc., Mukilteo, WA, USA). Transcutaneous carbon dioxide (TCM4/40, Radiometer, Denmark, Copenhagen), nasal pressure and oronasal airflow were also recorded. Oxygen saturation (SpO_2_) was measured using Bitmos GmbH (Bitmos, Dusseldorf, Germany), which uses Masimo signal extraction technology for signal processing and was set to a 2-second averaging time.

### Demographic information

Parents provided information regarding family structure, parental level of education, parental occupation, maternal age at child’s birth and English language exposure. Socio-economic status was determined using the Social Risk Index [38]. The Social Risk Index provides an indication of social and economic status based on six key aspects: family structure, highest education completed by primary care-giver, employment status of primary income earner, occupation of primary income earner, language spoken in the home, and maternal age at the birth of the child. The Social Risk Index is scored on a range of 0–12 with a higher score indicative of a lower socio-economic status. Maternal occupation was converted to an occupational status score developed from the Census of 2006 [39]. This score ranges from 0–100 with a higher score indicative of higher occupational prestige.

### Cognitive Assessment

The battery of cognitive testing was designed at baseline to assess the cognitive functions considered most at risk of deficit from symptoms of SDB. Details of the battery have been reported previously [6], however in brief, the Stanford-Binet Intelligence Scales, Fifth Edition [40] were used to provide an indication of global intellectual ability. The Abbreviated Battery IQ (ABIQ) was used in this instance, consisting of a measure of nonverbal reasoning (non-verbal IQ) and verbal knowledge (verbal IQ). Raw scores were converted into age-scaled standardized scores (M = 10, SD = 3) then summed and converted to a standardized ABIQ score (M = 100, SD = 15). The age-scaled standardized scores for each subset were also converted to IQ scores (M = 100, SD = 15) for analysis.

The NEuroPSYchological assessment (NEPSY) [41,42] was used to assess attention, language, visuospatial ability, and sensorimotor skills. Although the NEPSY is validated in children 3–16 years, it is designed to assess cognitive function at particular developmental milestones. As such not all subsets assessed at baseline could be compared to follow-up. In this study, the subscales assessing language comprehension (*phonological processing*), receptive language skills (*comprehension of instructions*), and visuospatial ability (*design copy*) were analyzed. Raw scores for each subset were converted into age-scaled scores (M = 10, SD = 3) and then into a standardized score (M = 100, SD = 15) for analysis.

### Behavioral Assessment

The CBCL 1.5–5 [43] (parent report) was used to assess problem behaviors and emotional difficulties at baseline. The CBCL 6–18 was used at follow-up [44]. The domain scores of the *Internalizing*, *Externalizing*, and *Total Problems Scales*, which comprise groups of the subscales, were compared across time. Due to developmental differences in behavior, not all sub-scales could be compared across the two time points. For this study, the *anxious/depressed*, *somatic complaints*, *withdrawn/depressed*, *attention problems*, and *aggressive behavior* sub-scales were analyzed.

The BRIEF (parent report) is designed to assess behaviors that directly reflect executive dysfunction. The Preschool version [45] was used at baseline and the 5-18y version [46] at follow-up. The *global composite score*, calculated from the scores on various subscales, was also in this study. The comparable subscales were *inhibit*, s*hift*, e*motional control*, *working memory* and *plan/organize*.

Scores for both the CBCL and BRIEF were converted into age-adjusted, normalized T-scores (M = 50, SD = 10) for analysis.

### Sleep Diary

Sleep/wake patterns for 7-days prior to the neurocognitive testing were assessed via parent-reported sleep diaries. A minimum of four consecutive days were required for inclusion in the analysis. These recorded what time the child went to bed, woke up and any night awakenings. Total sleep opportunity was calculated as the difference between bedtime and rise time less any night awakenings.

### Data Analysis

A minimum of 4 h of sleep on the night of the PSG was required for children to be included in the study (minimum recorded total sleep time was 5.6h at baseline and 5.4h at follow-up). PSG studies were manually sleep-staged in 30s epochs and respiratory events scored by experienced pediatric sleep technologists according to slightly modified AASM 2007 rules that were used clinically at the time of the follow-up studies [37]. Modification consisted of the inclusion of respiratory event related arousals (RERA) associated with arousal or desaturation into the obstructive apnea hypopnea index (OAHI) [47]. Baseline studies which were conducted prior to the laboratory updating these scoring criteria were rescored by two trained technicians who maintained a concordance rate >85% for both sleep and respiratory events. Obstructive apneas were defined as a >90% fall in airflow for ≥90% of event duration, with continued or increased respiratory effort. Mixed apneas consisted of a central component followed by an obstructive component. An hypopnea was associated with a ≥50% fall in airflow signal for at least ≥90% of the event, associated with an arousal, awakening or ≥3% desaturation. RERAs were scored where there was a 30% decrease in amplitude and flattening of the nasal pressure trace, associated with snoring, noisy breathing, elevation of the end-tidal or transcutaneous pCO_2_ and/or visual evidence of increased work of breathing, leading to an arousal from sleep or ≥3% desaturation.

Wake after sleep onset (WASO) was calculated as the percentage of time awake during the sleep period time (SPT), with SPT defined as the amount of time in minutes from sleep onset until lights on at the end of the study. Total Sleep Time (TST) was defined as SPT excluding all periods of wake. The OAHI was defined as the total number of obstructive apneas, mixed apneas, obstructive hypopneas and RERAs/h of TST. Criteria for the categorization of SDB severity mirrored current clinical practice: primary snoring (PS, OAHI ≤ 1 event/h); mild OSA (Mild OSA, OAHI between >1–5 events/h); or moderate/severe OSA (MS, OAHI >5 events/h). Other calculated variables included the respiratory disturbance index (RDI), sleep onset latency (SOL), sleep efficiency (SE), total arousal index (ARI), and SpO_2_ nadir. RDI was defined as the total number of scored respiratory events including obstructive apneas, mixed apneas, obstructive hypopneas, RERAs, central apneas and central hypopneas. SOL was defined as the time taken from lights out until the first epoch of N1 sleep and sleep efficiency was calculated as the percentage of the time asleep compared to the total time available for sleep. ARI was defined as the total number of cortical and subcortical spontaneous, respiratory and periodic leg movement arousals/h TST.

At follow-up, the children were divided into three groups according to whether their SDB had resolved (Controls, Resolved, Unresolved). SDB was considered resolved when the OAHI ≤ 1, there was no snoring reported on the night of the PSG, and the parents documented that they had not observed their child snoring loudly, holding their breath, making choking or gasping sounds during sleep, or being restless with frequent awakenings on the OSA-18 questionnaire.

### Statistical Analyses

Statistical analyses were performed using SPSS^®^ version 20 (IBM^®^, Chicago, IL). Data were first tested for normality and equal variance. SOL, WASO, and all respiratory parameters showed a positive skew. SOL and WASO were normalized using a logarithmic transformation [48]. Respiratory parameters could not be normalized, so were analyzed using non-parametric statistics. Group and time effects of continuous demographic data were analyzed using repeated measures analysis of variance (RM ANOVA). Group differences in categorical data were assessed using Chi-squared analysis. Group differences in sleep diary and PSG recorded sleep parameters over time were analyzed with RM ANOVA with post-hoc testing where appropriate (*p*<0.05). Differences in respiratory parameters were assessed using Kruskal-Wallis ANOVA on ranks, with Mann-Whitney U post-hoc testing where appropriate (*p*<0.05). Linear mixed-model analyses were used to determine the fixed effects of time and resolution of SDB on cognitive and behavioral outcomes. Correlational analysis revealed the Social Risk Index, maternal education, and maternal occupation to be significantly correlated with cognitive outcomes so were entered as covariates and accounted for in the model prior to the outcomes of interest. Time was entered into the model as the repeated measures component (Baseline and Follow-up) and as the first fixed effect. Separate analyses were conducted using RDI, OAHI, SpO_2_ nadir and ARI as covariates, with an interaction with Time included in the model, to determine the predictive value of changes in these parameters on changes in cognition or behavior. Subject was used as the random effect in this analysis, accounting for the inter-individual differences at baseline. Estimated margin of means comparisons were conducted for significant main effects of Group to determine the difference between Resolved, Unresolved and Control. Paired comparisons were conducted for significant main effects of Time (Baseline vs Follow-up).

## Results

Of the 191 children who participated in the baseline study, 36 could not be contacted, 76 declined further participation, two controls were ineligible as they were diagnosed with a behavioral disorder following the baseline study, and a further two controls were unable to undertake sleep studies in the available time period. Thirty percent of the clinically referred group and 75% of controls agreed to participate in the follow-up study and all underwent a repeat overnight PSG. Eight children from the clinically referred group did not participate in the cognitive assessment component of the study and one was excluded following the cognitive assessment due to incomplete data. Two children from the control group were subsequently excluded from analysis as they had developed SDB. There were no differences in any of the demographic variables, sleep parameters or cognitive and behavioral outcomes at baseline between control children who did and those who did not return.

Of the 76 children who refused further participation, 58 (76%) had complete cognitive and behavioral data at baseline so were eligible for testing at follow-up. Of those eligible but who declined to participate, 49% stated their reason for refusal to be that the original referral issue had resolved and they did not feel their child needed a repeat sleep study. Other reasons for refusal included: the child refused to participate (18%); parent was too busy (12%); parental illness or accident (7%); parent was unhappy with the clinical process at baseline (7%); or the family had moved outside the metropolitan area (7%). Seven percent did not provide a reason for refusal.

Following the baseline study, approximately half (56%) of children with SDB who did not return for the follow-up were not treated. Of those that were treated, adenotonsillectomy was the most common treatment (86%) with the remainder treated with nasal steroids (14%). The proportion of children who were treated was not significantly different between the children who participated and those who did not (*p* = 0.15). There were also no differences in age, gender, BMI-Z score, maternal occupation, or maternal education between those that did and those that did not return for the follow-up study. The Social Risk Index was significantly higher in those who did not return (M±SD = 1.4±1.7) compared to those who did return (M±SD = 0.8±1.1, *p* = 0.02), indicating that those who refused were from a slightly lower socio-economic background. There were no differences at baseline in any of the cognitive, behavioral or sleep outcomes between the children with SDB who did and did not return.

**Fig 1:**
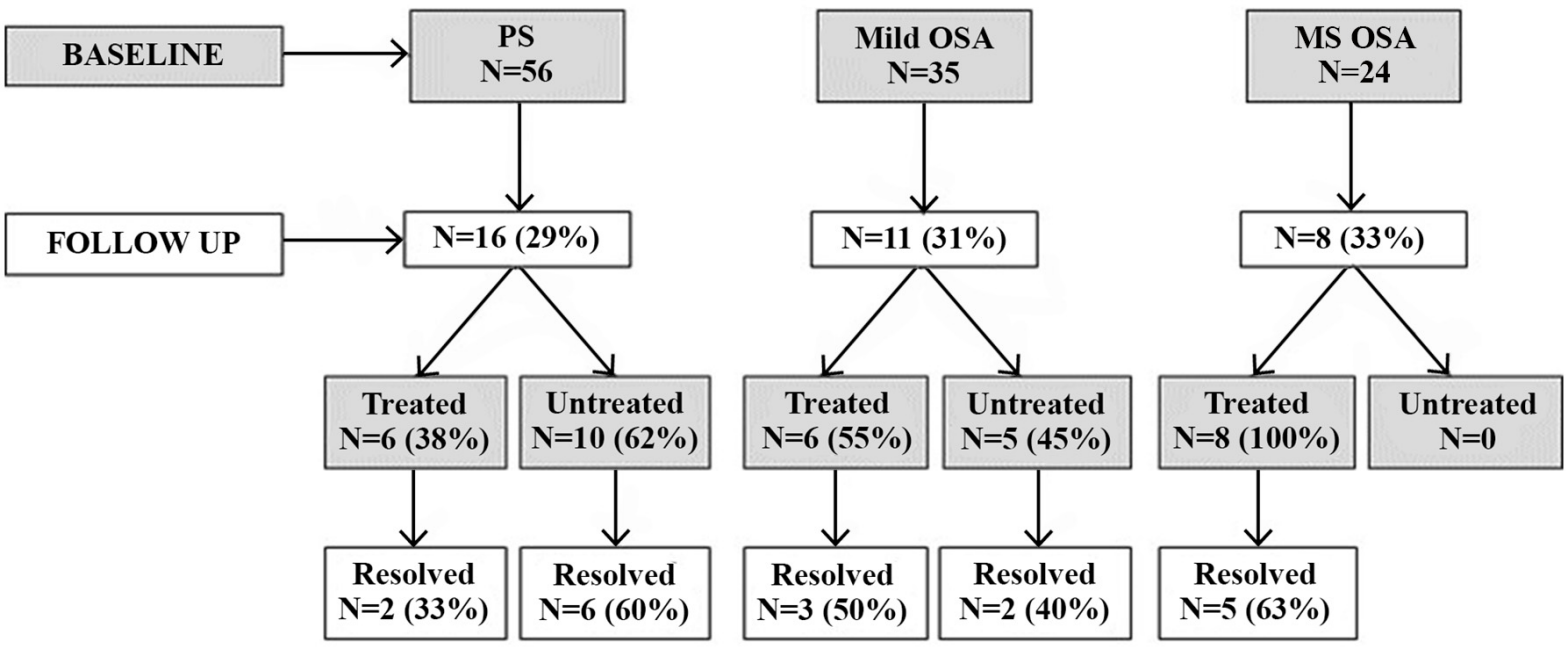
Profile of SDB cohort at baseline and follow-up. At follow-up 16 children originally diagnosed with PS, 11 with Mild OSA and 8 with MS OSA returned. Of these, 18 had resolved and 17 had ongoing SDB. Twelve children who had resolved received treatment. Eight children who were unresolved also received treatment.

The follow-up profile of the returning children diagnosed with SDB at baseline is presented in Fig 1. In summary, of the children whose SDB resolved, 56% received treatment and 44% resolved spontaneously. Of the children with unresolved SDB, 59% were treated and 41% were untreated.

### Demographics

Demographic data are presented in Table 1. There were no differences in age, BMI z-score, or exposure to English language between the 3 groups. There were significantly more males in the resolved group. The percentage of mothers with a tertiary education was lowest in the Resolved group, which was also reflected in the lower maternal occupation score and higher score on the Social Risk Index.

**Table 1:** Cohort demographics.

	Control (N = 25)		Resolved (N = 18)		Unresolved (N = 17)		*P*
	Baseline	Follow-up	Baseline	Follow-up	Baseline	Follow-up	
Age (Years: Mean±SD)	4.2 (1.1)	7.5 (1.1)	4.6 (0.8)	7.8 (0.9)	4.2 (0.8)	7.5 (1.2)	NS^a^
BMI-Z score (Mean±SD)	0.5 (1.2)	0.4 (0.9)	0.7 (1.2)	0.7 (0.8)	0.6 (1.0)	0.3 (1.0)	NS^a^
Gender (N, % Male)		11 (44)		15(83)		8 (47)	0.02^b^
Maternal Education (N, % Tertiary)		18 (72)		7 (39)		12 (75)	0.03^b^
Maternal Occupation (Mean±SD)		74 (19)		58 (17)		67 (19)	0.02^c^
English Language Exposure (N, % English Only)		23 (92)		11 (61)		11 (65)	NS^b^
Social Risk Index (Mean±SD)		0.4 (0.9)		1.2 (1.5)		0.9 (1.1)	0.02^d^

^a^ Repeated Measures ANOVA—significance value of group main effect reported;

^b^ Chi-squared analysis;

^c^ One-way ANOVA;

^d^ Kruskal-Wallis ANOVA on ranks

### Sleep Characteristics

The changes in sleep diary and PSG sleep parameters over time for each group are shown in Table 2. The amount of total sleep opportunity as reported by parents did not change over time or between groups. As is expected with increasing age, the percent of N1 and REM decreased and N2 and total non-REM increased over time. The percent of WASO also increased over time, resulting in decreased sleep efficiency between baseline and follow-up. Overall group differences were found in the percent of total non-REM and REM with the Control group showing significantly more non-REM and less REM compared to the Resolved group. There were no significant interactions between group and time indicating that the change in sleep parameters over time was equivalent between groups.

**Table 2:** Results of repeated measures analysis for sleep variables. Data are presented as mean(±SD).

	Control		Resolved		Unresolved		Time	Group	Time x Group
	Baseline	Follow up	Baseline	Follow up	Baseline	Follow up	F	F	F
**Sleep Diary– 4–7 days**									
TSO (minutes)	635 (56)	613 (38)	619 (32)	614 (40)	611 (48)	593 (48)	2.6	1.5	0.3
**Sleep Parameters PSG**									
SOL (minutes)	25 (25)	27 (29)	18 (11)	25 (28)	25 (30)	28 (17)	0.4	0.1	0.3
TST (minutes)	451 (38)	430 (43)	442 (35)	421 (58)	435 (38)	437 (42)	3.5	0.4	1.1
WASO (% of SPT)	7 (5)	10 (6)	6 (4)	12 (8)	7 (7)	8 (6)	9.4**	0.7	1.4
REM Latency (minutes)	113 (46)	141 (59)	111 (45)	187 (74)	123 (47)	140 (53)	14.5***	1.9	2.8
N1 (% of TST)	8 (4)	7 (3)	8 (4)	6 (2)	10 (5)	7 (3)	10.1**	1.4	0.2
N2 (% of TST)	41 (5)	47 (6)	42 (6)	46 (6)	40 (8)	46 (8)	37.1***	0.1	0.4
N3 (% of TST)	27 (5)	27 (5)	30 (6)	30 (7)	30 (6)	26 (5)	2.7	2.7	1.7
Total NREM (% of TST)	76 (5)	80 (4)	81 (3)	82 (4)	80 (3)	80 (5)	5.6*	5.3** ^a^	2.3
REM (% of TST)	23 (5)	20 (4)	19 (3)	18 (4)	20 (3)	20 (5)	4.9*	4.9* ^b^	1.9
SE (%)	88 (7)	85 (8)	90 (5)	82 (8)	87 (10)	87 (5)	6.3*	0.2	2.3

* *p*<0.05,

** *p*<0.01,

*** *p*<0.001.

^a^ Control < Resolved;

^b^ Control > Resolved

TSO = Total sleep opportunity; SOL = Sleep onset latency; TST = Total sleep time; WASO = Wake after sleep onset; SPT = Sleep period time; NREM = Non-Rapid Eye Movement, REM = Rapid Eye Movement; SE = Sleep efficiency

### Respiratory Parameters

The changes in respiratory parameters on PSG over time for each group are shown in Table 3. By design, significant differences in RDI and OAHI between the Control and Resolved groups at baseline were no longer evident at follow-up. RDI and OAHI remained significantly higher in the Unresolved group compared to the Control group and became significantly higher than the Resolved group at follow-up. This pattern was also observed in the arousal index. The SpO_2_ nadir improved for all groups over time.

**Table 3:** Results of non-parametric analysis for PSG respiratory variables. Data are presented as median(interquartile range).

	Control		Resolved		Unresolved		Group^a^		Time^b^
	Baseline	Follow up	Baseline	Follow up	Baseline	Follow up	χ^2^ Baseline	χ^2^ Follow up	Z
**Respiratory Parameters**									
RDI (events/h)	0.8 (0.7–1.9)	1.0 (0.5–1.8)	4.4 (1.5–9.1)	0.8 (0.8–1.5)	3.5 (2.7–6.5)	3.1 (1.8–4.4)	21.6***	18.9***	-2.8**
OAHI (events/h)	0.0 (0.0–0.1)	0.0 (0.0–0.4)	1.5 (0.2–6.9)	0.1 (0–0.4)	1.4 (0.6–3.8)	2.4 (0.7–3.1)	26.3***	21.3***	-1.7
SpO_2_ nadir (%)	93 (91–95)	94 (93–96)	92 (88–95)	95 (93–96)	93 (86–95)	94 (92–96)	0.4	1.3	-3.9***
ARI (events/h)	10.1 (9.0–14.7)	9.8 (7.7–12.6)	14.5 (11.1–18)	10.3 (8.6–13.2)	14.5 (10.9–20.1)	12.2 (9.5–14.7)	9.6**	4.6	-3.4**

** *p*<0.01,

*** *p*<0.001.

^a^ Group differences at each time point analysed using Kruskal-Wallis Test.

^b^Changes in respiratory outcomes between Time 1 and Time 2 analysed using Wilcoxon Signed Rank Test

RDI = Respiratory disturbance index; OAHI = Obstructive apnea hypopnea index; CRDI = Central respiratory disturbance index; REM RDI = percentage of respiratory events in REM; SpO_2_ nadir = minimum oxygen saturation; SpO_2_ dips >4% = number of oxygen saturation dips/h >4%; ARI = Arousal index; RES-ARI = percentage of respiratory arousals

### Cognition

**Fig 2:**
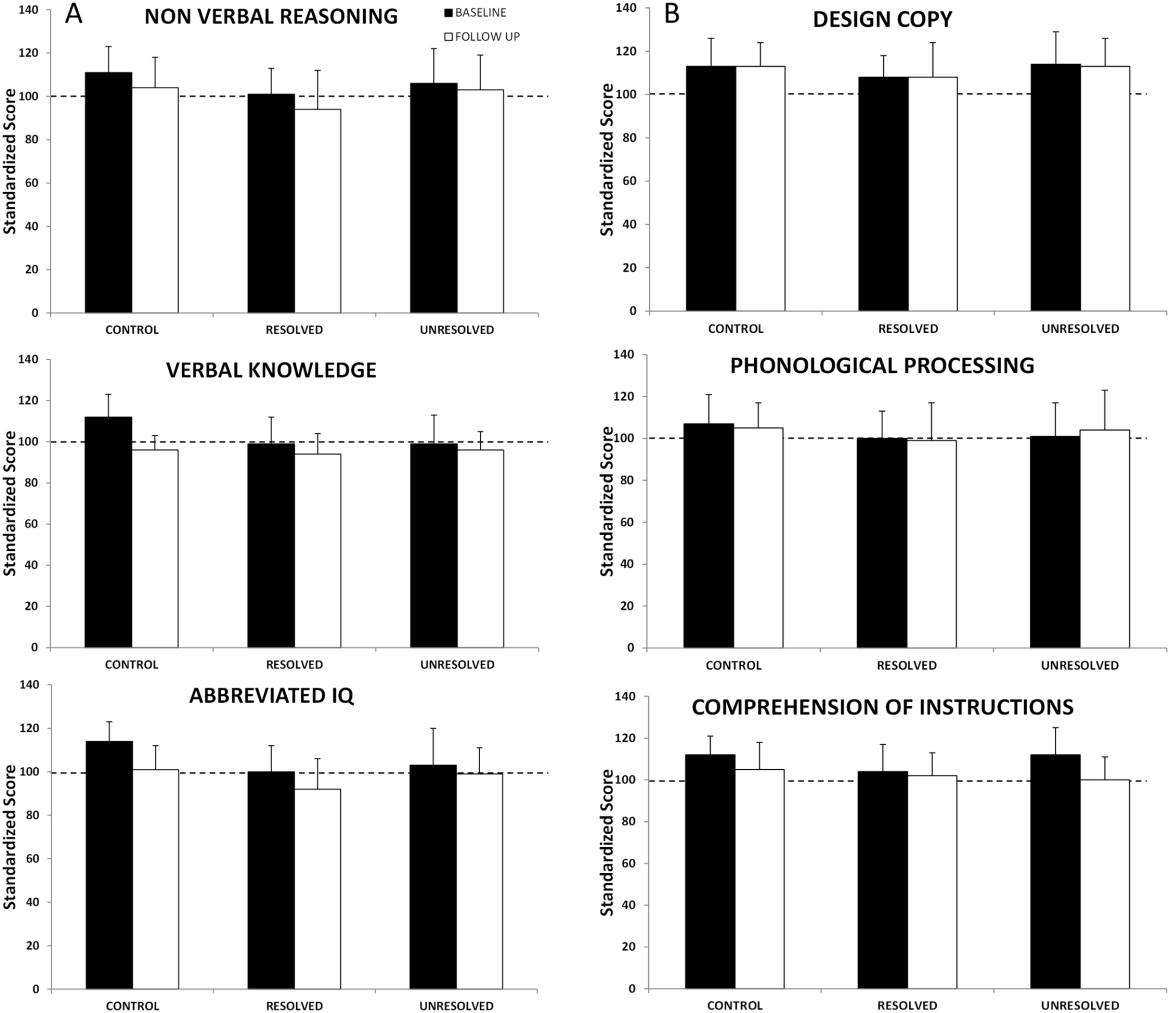
Mean scores on Stanford-Binet (2A) and NEPSY (2B) at baseline (black bars) and follow-up (white bars) for the Control, Resolved and Unresolved groups. The dotted line represents the population mean.

The mixed model analysis revealed a significant effect of time for all IQ measures, with scores reducing on verbal knowledge, nonverbal reasoning, and abbreviated IQ between baseline and follow-up in all three groups. Means (±SD) are presented graphically in Fig 2A. There were no group differences on any of the IQ measures.

There was also a significant main effect of time for the NEPSY comprehension of instructions task (F_(1,61.5)_ = 5.3, *p*<0.05), with age scaled scores reducing from baseline to follow-up (Fig 2B). No group effects were found. There were also no effects of time or group on either of the other two NEPSY subtests: design copy and phonological processing (Fig 2B).

Despite significant correlations between the Social Risk Index, maternal education, maternal occupation and cognitive outcomes, these factors did not predict scores on cognitive measures over time with no significant effects observed in the mixed model.

As can be seen by the model estimates in Table 4, OAHI, SpO_2_ nadir, and ARI were not related to overall performance on any cognitive task. There were also no interaction effects between these variables and time, indicating that any change in cognitive scores was not related to changes in OAHI, SpO_2_ nadir, or number of arousals. There was a significant negative effect of RDI on the comprehension of instructions score, suggesting that a higher RDI was associated with a lower score in the comprehension of instructions task. The mixed model also revealed a significant interaction effect between time and RDI (F_(1,79.5)_ = 4.6, *p*<0.05), indicating that a change in RDI was associated with a change in scores in this task.

**Table 4:** Mixed model estimates of fixed effects of respiratory parameters on cognitive and behavioral outcomes.

	PSG Respiratory Parameters			
	RDI	OAHI	SpO_2_ nadir	ARI
	β (SE)	β (SE)	β (SE)	β (SE)
**Stanford Binet**				
Verbal Knowledge	-0.5 (0.7)	0.2 (0.8)	-0.9 (0.5)	-0.6 (0.4)
Nonverbal Reasoning	0.1 (1.1)	1.0 (1.3)	1.1 (0.7)	-0.4 (0.6)
Abbreviated IQ	-0.0 (0.8)	0.8 (0.9)	0.3 (0.5)	-0.4 (0.4)
**NEPSY**				
Design Copy	-2.2 (1.2)	-0.9 (1.4)	0.4 (0.9)	-1.0 (0.6)
Phonological Processing	-1.8 (1.2)	-1.0 (1.4)	0.6 (0.8)	-0.6 (0.6)
Comprehension of Instructions	-1.8 (0.8)*	-1.7 (0.9)	0.6 (0.5)	-0.7 (0.4)
**CBCL**				
Internalizing Scale	-1.0 (0.5)	-1.5 (0.6)*	0.2 (0.4)	-0.1 (0.3)
Externalizing Scale	0.3 (0.5)	-0.1 (0.6)	0.2 (0.4)	0.2 (0.3)
Total Problems Scale	-0.4 (0.5)	0.7 (0.6)	0.1 (0.5)	0.1 (0.3)
Anxious/Depressed	0.2 (0.4)	-0.2 (0.4)	-0.0 (0.3)	0.3 (0.2)
Somatic Complaints	-0.2 (0.4)	-0.4 (0.5)	0.1 (0.3)	-0.2 (0.2)
Withdrawn/Depressed	-0.1 (0.4)	-0.5 (0.4)	0.3 (0.3)	0.0 (0.2)
Attention Problems	0.7 (0.3)*	0.5 (0.4)	-0.2 (0.3)	0.7 (0.2)***
Aggressive Behaviour	0.9 (0.4)*	-0.5 (0.4)	-0.1 (0.3)	0.5 (0.2)*
**BRIEF**				
Global Executive Composite	0.8 (0.5)	0.9 (0.6)	-0.1 (0.4)	0.6 (0.2)*
Inhibit	0.5 (0.5)	0.5 (0.5)	-0.5 (0.4)	0.6 (0.2)*
Shift	0.3 (0.5)	0.3 (0.6)	0.5 (0.4)	0.3 (0.3)
Emotional Control	0.6 (0.7)	0.3 (0.8)	-0.1 (0.5)	0.8 (0.3)*
Working Memory	1.1 (0.7)	1.1 (0.8)	-0.2 (0.5)	0.7 (0.3)
Plan Organise	1.1 (0.6)	1.2 (0.7)	-0.2 (0.5)	0.4 (0.3)

* *p*<0.05,

*** *p*<0.001

### Behavior

**Fig 3:**
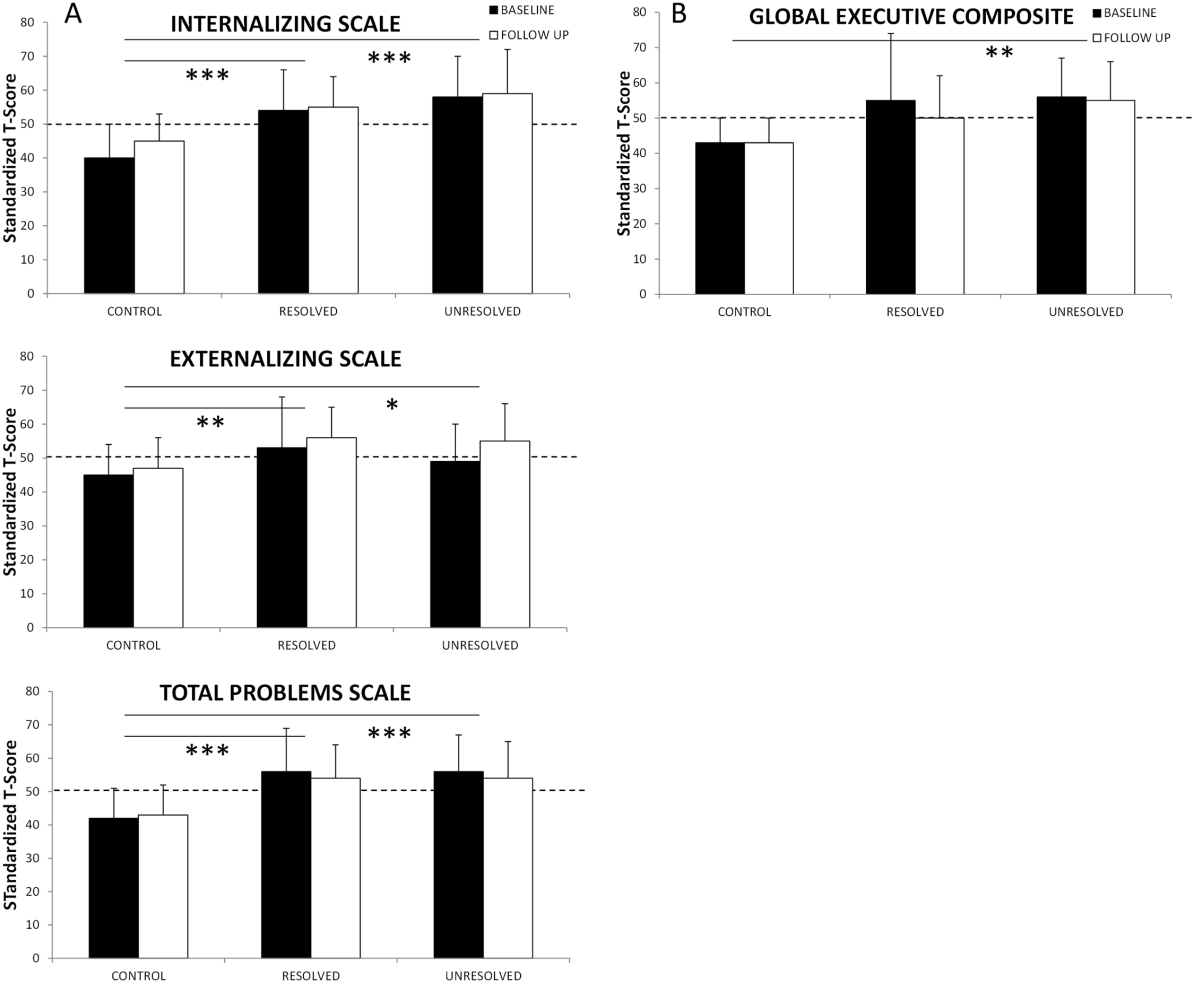
Mean domain scores on CBCL (3A) and BRIEF (3B) at baseline (black bars) and follow-up (white bars) for the Control, Resolved and Unresolved groups. The dotted line represents the population mean. The solid lines represent the between group differences. *p<0.05, **p<0.01, ***p<0.001.

Means (±SD) for scores on the CBCL domains of internalizing, externalizing and total problems are shown in Fig 3A. The between group differences are indicated by the horizontal lines. Again, there was no effect of time, but significant group differences in all domains were observed with parents of the Control group reporting better behavior than either the Resolved or Unresolved groups.

Mixed model analysis showed no effect of time on any of the CBCL subscales, suggesting behavior remained unchanged between baseline and follow-up. There were significant main effects for group for the CBCL subscales of anxious/depressed (F_(2,55.3)_ = 13.6, *p*<0.001), somatic complaints (F_(2,58.8)_ = 15.2, *p*<0.001), withdrawn/depressed (F_(2,59.1)_ = 9.3, *p*<0.001), attention problems (F_(2,56.1)_ = 4.0, *p*<0.05), and aggressive behavior (F_(2,55.7)_ = 3.5, *p*<0.05). Post-hoc analyses revealed that for the anxious/depressed and withdrawn/depressed subscales, there were no differences between the Resolved and Unresolved groups, however both groups scored significantly higher (i.e. more behavior problems) than the Control group. For somatic complaints, scores were not different between the Control and Resolved groups, with both groups scoring significantly lower (i.e., fewer somatic complaints) than the Unresolved group. There were significantly fewer attention problems reported in the Control group compared to the Resolved group. Group differences did not reach statistical significance in post-hoc testing of aggressive behavior.

As Table 4 shows, OAHI was negatively associated with internalizing problems, indicating that a lower OAHI was predictive of more concern in this domain. The interaction with time was also significant. RDI and ARI were positively associated with attention problems and aggressive behavior, suggesting that a higher RDI and/or ARI was predictive of more problems in these behaviors. There was also a significant interaction between these respiratory variables and time, indicating that a change in RDI and/or ARI was directly related to a change in scores on the attention problems and aggressive behaviors sub-scales. This relationship was particularly strong between ARI and attention problems (F_(1,62.8)_ = 11.1, *p* = 0.001).

The means (±SD) for the global executive composite are shown in Fig 3B which shows overall significant group differences between the Unresolved and Control group. Overall group differences were found in shift (F_(2,56.4)_ = 12.4, *p*<0.001), emotional control (F_(2,58.0)_ = 7.9, *p* = 0.001), working memory (F_(2,57.2)_ = 6.5, *p*<0.01), and plan/organize (F_(2,57.6)_ = 4.4, *p*<0.05). For all subscales except plan/organize, post-hoc comparisons showed that parent ratings for the Control group indicated significantly better executive functioning than both the Resolved and Unresolved, with no differences between the SDB groups. In plan/organize, the Control group rating was significantly lower (i.e. better functioning) than the Resolved group only.

As Table 4 shows, there were no effects of RDI, OAHI, or SpO_2_ nadir on the BRIEF composite scale or any of the subscales. The ARI was positively associated with the global executive composite scale and the inhibit and emotional control subscales, indicating that the greater the sleep fragmentation, the worse the reported executive skills in these areas. There was no significant interaction with time, suggesting that any changes in the arousal index were not directly related to any changes in these executive functions.

## Discussion

The results of this longitudinal study suggest that resolution of SDB, as defined by an OAHI≤1 and absence of snoring, in preschool children has little effect on cognitive or behavioral outcomes in the long term. Overall, irrespective of whether SDB resolved or there was ongoing disease, cognitive performance reduced or remained unchanged. Behavioral functioning remained significantly worse in children originally diagnosed with SDB than observed in non-snoring peers. This does not imply that treatment should be abandoned. There is sound evidence that treatment improves nocturnal respiration and sleep, as was demonstrated in this study. These improvements, particularly improvements in nocturnal arousals, were associated with some aspects of behaviour. Treatment also improves cardiovascular outcomes in older children [11,13,49], perhaps more clinically significant than small decrements in cognition and behavior. Yet, these results do call into question the suggestion that the ongoing cognitive and behavioral deficits observed in later childhood following treatment are solely the result of residual disease [22].

Sixty percent of the children who received treatment had resolution of SDB and 40% of those who did not receive treatment had spontaneous resolution. Conversely, 59% of children with on-going disease had received treatment. Although there was a change in cognitive performance over the three years of this study, the direction was not as expected. All three groups showed decline in scores on IQ and language comprehension, which was not related to any change in physiological measures of SDB. Behavior remained of greater concern to parents of children originally diagnosed with SDB compared to non-snoring control children, irrespective of whether their SDB had resolved. Overall, sleep fragmentation, depicted by the number of arousals during sleep, showed the greatest association with behavioral scores, with improvements in the arousal index predicting improvements in attention problems and aggressive behavior.

As the reduction in cognitive performance was observed in the non-snoring control group as well as the SDB groups, possible methodological or ecological explanations must be considered. The decrease in scores may be an artifact of the psychometric scaling. With age scaled normative scores, the standardized IQ score of a child can reduce by up to six points simply by moving from one age bracket (e.g. 3y11m) to another (4y0m), creating systematic bias [50]. However, while this presents an issue for retesting in the short term, it is unlikely to be the explanation for the current results. Based on the normative data it would be expected that there would be an increasing trend in mean raw scores until late adolescence [51]. In the current study, mean raw scores decreased in all groups (results not reported). It may be that the children experienced a greater sense of overall fatigue at follow-up as they had entered school, which would have changed the intensity of their activities and limited the opportunity for napping patterns [52]. Furthermore, all assessments for the follow up study were conducted on Saturdays, whereas baseline studies were routinely conducted on weekdays. This may have had a direct impact on motivation and effort as children may be less inclined to do “school work” on weekends.

Although the reduction in cognitive performance in the Resolved group over time was surprising, it is not unprecedented. Giordani et al. [22] also found a reduction in performance on cognitive tasks, particularly those involving verbal ability, one year after adenotonsillectomy. In that study, children who underwent adenotonsillectomy for OSA and a comparison group who underwent adenotonsillectomy for other medical reasons both showed significant reductions in aspects of verbal ability, verbal learning, and numerical operations. These authors suggest that the results were possibly due to residual disease, early insult to the developing brain, or that deficits appear phenotypically despite early interventions. Using our definition of SDB resolution, on average, the children with OSA (aged 5–12 y) in that study would have been considered to have resolved SDB following surgery (mean obstructive apnea index = 0.18 events/h, no evidence of snoring).

Our earlier study examining the long-term outcomes of treatment in school-aged children with SDB reported little recovery of cognitive functioning and no improvement in behavioral functioning, irrespective of whether the children were treated or not [18]. However, 14% of that cohort who received treatment did not have full resolution of their SDB (defined as OAHI<1 event/h, no reports of habitual snoring) and 34% who did not receive treatment did experience full resolution spontaneously. Similar results were found by the Childhood Adenotonsillectomy Trial (CHAT) which studied treatment effects on sleep, respiration, behavior, cognition, and quality of life in a large cohort of 464 school-aged children (5–9 y) with mild OSA (apnea hypopnea index (AHI) >2 events/h), randomly allocated to early adenotonsillectomy or watchful waiting (no treatment) [13]. In that study, 21% of the children in the early adenotonsillectomy group did not have full resolution of their OSA (AHI >2 events/h following surgery), and almost half (46%) of children in the watchful waiting group had spontaneous resolution of their OSA (AHI<2 events/h) despite no intervention. Huang and colleagues [31] also found that not only did OSA not resolve in a proportion of school-aged children following treatment, but in some cases, symptoms became progressively worse 12, 18, and 36 months following surgery. The results of current study suggests that the reduction in cognitive performance may not be the result of residual disease as both the resolved and unresolved groups had lower scores on cognitive tests over time. It does not, however, rule out irreparable damage due to early insult to the developing brain. Our study did not assess how long the children had been snoring prior to their first diagnostic PSG at age 3–5 years. A birth-cohort study would elucidate whether snoring before 3 years of age has irrecoverable effects on cognitive development.

The decision to analyze our cohort depending on whether their SDB had resolved or not, rather than simply whether they had been treated, and the lack of any cognitive or behavioral changes lends support to Giordani et al.’s postulation that the cognitive and behavioral profile of SDB may be phenotypically driven [22]. As has been suggested previously [53], it may be that there is an underlying gene-related susceptibility that is mediating the relationship between SDB and cognitive and behavioral morbidity. If this were the case, deficits could appear irrespective of early interventions. The existence of a SDB phenotype cannot be fully addressed by our study, however the results highlight the need for further exploration.

The stability of behavioral problems in children with SDB following treatment in our study is contrary to what is reported in treatment studies with shorter follow-up periods [10,13,14], however is consistent with our previous longitudinal study of school aged children with SDB [18]. The discrepancies with shorter term studies may be due to an acute response to treatment, with parental perceptions of behavior in the short-term reflective of improvements in sleep, rather than a global behavior change [54]. Two studies have reported parent-reported behavioral outcomes in children immediately following treatment of OSA (≤6mths) and then at varying intervals up to 4-years following surgery [14,55]. The longitudinal profile in both these studies showed a worsening of behavioural outcomes over time when compared to immediate post-treatment assessments, although deficits did not return to pre-surgery levels.

Alternatively, the stability of behavioral problems may reflect an initial referral bias in that parents may have sought a medical explanation for their child’s behavioral problems and the fact that the child snored provided such an explanation. Once treated, the expectation of improvement may have biased parents responses to the behavioral questionnaires in the short term [56]. As time progresses and the memory of pre- and immediate post-surgery sleep and behavior fades, behavioral habits and parental responses to problem behaviors may once again become salient.

The counter-intuitive negative predictive relationship observed between OAHI and Internalizing Behavior is difficult to explain. In our publication of the full cohort at the time of the baseline study[6], we noted an unexpected finding, namely children with PS were rated as having significantly poorer behavioural functioning than children with MS OSA. It may be that the negative association between OAHI and Internalizing Behavior is a reflection of the fact that children with PS were less likely to resolve, as per our definition, than children with MS OSA in this study, yet continued to have greater scores on the Internalizing Behavior sub-scale. As we cannot perform statistical analysis on individual groupings due to the small sample size, we can only postulate, however the distribution of mean scores as shown on Fig 3 appears to support this.

The predictive relationship between the change in the arousal index and a number of behavioral outcomes suggests that the association between sleep fragmentation and behavior is not dependent on SDB, but may be strengthened by it. The current results indicate that an improvement in the number of arousals was predictive of an improvement in attention and aggressive behavior which was independent of whether there was resolution of SDB (as per our definition). Numerous studies have been unable to establish a predictive association between PSG parameters and cognitive and behavioral outcomes, either prior to or following treatment [19,21,57–59], resulting in an ongoing debate regarding the sensitivity of the current assessment measures used to define SDB. Indeed, although the arousal index in the current study showed a statistically significant improvement over time, the magnitude of the actual change seems unlikely to be of clinical significance. Mean number of arousals per hour for the entire cohort reduced from 13.8 to 11.2. This is similar to the 14% decrease reported by Chervin et al. [60] who found no association between sleep fragmentation, defined by respiratory cycle-related changes in the EEG, and neurobehavioral outcomes following treatment. The mixed model analysis used in the current study may have provided the statistical sensitivity to detect an association, however the clinical relevance needs to be considered. Therefore, while this is a promising result regarding a potential mechanistic pathway between SDB and daytime deficits, these results do need to be interpreted with caution.

The primary limitation of this study was the low response rate for the follow-up study, with those refusing outnumbering those who agreed to participate. Although comparison of the two groups revealed similar demographic backgrounds and treatment history, there may be a selection bias involved as half of those who refused to return were under the impression that their child’s SDB had resolved. The small sample as a result of the low response rate may have also resulted in the study being underpowered to observe a statistical difference in the outcomes. It also precluded our ability to further explore the differences between children who were treated and had resolved versus those who were treated and had not resolved. It must be acknowledged that as the decision to treat was based on clinical practice and not randomized, the children with SDB who were treated did have more severe disease at baseline than those who were not treated. Further examination of the specific characteristics of those children who do not respond to treatment is an important area for further investigation. However, the use of linear mixed modelling has the advantage over univariate models in that there is no assumption of homogeneity or of independence. [61] As both within and between subject variance is accounted for in the model, we are confident that that are true results, despite the small sample. Regression towards the mean cannot be excluded when considering the pattern of cognitive results over time [62]. Although the psychometric testing is considered objective, it was impossible to blind parents and the psychologist (SB), who was involved in the study at baseline, to the treatment status of the children. However, whether the SDB of an individual child had resolved was unknown to the psychologist prior to conducting the cognitive assessments and thus any bias would have negligible impact on the current analysis. Although standardise testing protocols were used, an evaluation of concordance at both time points would have been useful to further assess bias. Finally, it must be acknowledged that the sleep studies were only conducted on a single night at both time points. Previous research in adults has shown that night-to-night consistency of OAHI is more accurate in those with more severe disease [63,64], but this is not the case in children. [65] While there may be some variation in OAHI across multiple nights, it is unlikely to have affected the clinical diagnosis.

## Conclusion

The current results suggest that resolution of SDB, as per our definition, in preschool children has little effect on cognitive or behavioral outcomes over the long term. Overall, irrespective of resolution of SDB or ongoing disease, cognitive performance reflected that observed in non-snoring peers and behavioral functioning remained significantly worse in comparison. While treatment, whether surgical or otherwise, should always be considered due to the proven physiological and health benefits, the effects on cognitive or behavioral deficits associated with SDB may be disappointingly small. Although not definitive, this study supports the possibility of a SDB phenotype. It also supports the contribution of disturbed sleep to behavioural problems whether related to SDB or not, highlighting the need for future targeted research.
